# Inflammatory Cytokines Protect Retinal Pigment Epithelial Cells from Oxidative Stress-Induced Death

**DOI:** 10.1371/journal.pone.0064619

**Published:** 2013-05-21

**Authors:** Helene B. Juel, Carsten Faber, Signe G. Svendsen, Abbe N. Vallejo, Mogens H. Nissen

**Affiliations:** 1 Eye Research Unit, Department of International Health, Immunology and Microbiology, University of Copenhagen, Copenhagen, Denmark; 2 Department of Pediatrics, Children's Hospital of Pittsburgh, Department of Immunology, Pittsburgh Cancer Institute, and McGowan Institute for Regenerative Medicine, University of Pittsburgh School of Medicine, Pittsburgh, Pennsylvania, United States of America; Duke University, United States of America

## Abstract

**Purpose:**

To investigate the effects of inflammatory factors and oxidative stress on cell survival of the human retinal pigment epithelial (RPE) cell line, ARPE-19.

**Methods:**

Confluent RPE cells were treated with peripheral blood mononuclear cells-conditioned medium (PCM), H_2_O_2_, NaIO_3_, interferon (IFN)-γ, tumor necrosis factor (TNF)-α, or combinations of these. Cell viability was determined by viability assays and by light microscopy. Effector molecules of cell death were investigated by immunofluorescence microscopy and flow cytometry. Microarrays were performed to screen for differential expression of anti-oxidative enzymes, and protein expression was validated by immunoblotting.

**Results:**

Viability of RPE cells was reduced by exposure to inflammatory agents (PCM, IFNγ+/-TNFα) or to oxidative agents (H_2_O_2_ or NaIO_3_). Unexpectedly, cells treated with either H_2_O_2_ or NaIO_3_ were partially protected from cell death by the addition of PCM. This protection was conferred, at least in part, by IFNγ and TNFα. Cell death induced by H_2_O_2_ or NaIO_3_ was preceded by mitochondrial dysfunction and by p62 upregulation, both of which were attenuated by PCM and/or by IFNγ+TNFα. RPE cells co-cultured with activated T cells, or treated with cytokines showed increased expression of anti-oxidative genes, with upregulation of superoxide dismutase 2 protein following PCM treatment.

**Conclusion:**

Oxidative stress-induced cell death was reduced by concomitant inflammatory stress. This is likely due to the cytokine-mediated induction of the anti-oxidative stress response, upregulating protective anti-oxidant pathway(s). These findings suggest caution for the clinical use of anti-inflammatory agents in the management of immune-associated eye diseases such as age-related macular degeneration.

## Introduction

The retinal pigment epithelium (RPE) constitutes the outermost layer of the retina, and has many important functions in the homeostasis of the eye to maintain normal vision. The RPE is under constant pressure from high metabolic demands in an environment with high levels of oxidative stressors. Dysfunction of the RPE has detrimental consequences as it precedes photoreceptor atrophy in several eye diseases, including age-related macular degeneration (AMD) [1].

AMD is a leading cause of vision loss [2], but there is still no consensus about the initiating event(s) or biochemical pathway(s) that result in clinical AMD disease. Recent experimental studies point towards immunological and oxidative processes that lead to RPE cell death [3].

Drusen, the sub-RPE deposits that are the hallmark of AMD, contain many inflammatory proteins including complement factors [4]–[8], cytokines [9], C-reactive protein [7], [10], IgG [11], and major histocompatibility class II molecules [5], [7]. It has been suggested that the accumulating drusen trigger local production of inflammatory mediators, and attract leukocytes that would in turn lead to an increase in local inflammation and retinal stress [12]. It has been reported that elevated levels of circulating leukocytes [13]. or inflammatory molecules [14]–[17] increase the risk of AMD. Hence, several anti-inflammatory drugs including complement inhibitors, tumor necrosis factor (TNF)-α inhibitors, and dexamethasone are currently on clinical testing for use in AMD [18]–[21].

The role of oxidative stress in the pathophysiology of AMD is widely acknowledged. The retina and RPE is highly exposed to oxidative stress due to the high metabolism, high exposure to scattered light, lipofuscin content, and hypoxia, all of which contribute to the generation of reactive oxygen species (ROS) [22], [23]. Several studies have reported lowered expression of anti-oxidative enzymes such as catalase and heme oxygenase 1 in the RPE, correlating with age or with incident AMD [24], [25]. Dietary antioxidant supplements have also been reported to reduce the risk of AMD progression, and are currently the only available treatment for atrophic AMD [26]–[28].

Autophagy is an intracellular process involved in protein degradation by the lysosomal pathway [29], and is used by cells during times of low nutrient levels, for degradation of damaged proteins or organelles, and for elimination of intracellular pathogens [30]. Autophagy is present at a basal level in healthy cells, and becomes upregulated under conditions of hypoxia, oxidative stress, and inflammation [31]. Depending on the cell type, efficiency of autophagy diminishes with chronologic aging [32]. Though often described as a cellular death mechanism because of its up-regulation in dying cells [33], [34], autophagy is also a cell survival strategy to withstand stress, and to reduce toxic effects of protein aggregates or damaged organelles [35].

It is probable that defects in autophagy contribute to AMD pathogenesis. Several autophagy-related proteins and exosome markers have been found in drusen [36], [37]. With advancing age, the RPE accumulates intracellular granules containing oxidized lipids, termed lipofuscin [38]. This has been associated with AMD pathogenesis, and lipofuscin is likely composed of indigestible waste products from inefficient autophagy [39].

In this study, we investigated responses of RPE cells treated with inflammatory stressors (peripheral blood mononuclear cells-conditioned medium (PCM), interferon (IFN)-γ, and TNFα), oxidative stressors (H_2_O_2_ and NaIO_3_), or a combination of these. We found that inflammatory or oxidative stressors alone caused RPE cell death, and hypothesized an additive or synergistic effect. Unexpectedly, inflammatory factors protected RPE cells against oxidative stress-induced cell death, which was likely caused by induction of 1) protective autophagy, and/or 2) anti-oxidative stress response(s).

## Materials and Methods

### Ethics statement

Peripheral blood mononuclear cells (PBMCs) were purified from fresh whole blood from healthy, young volunteers. Verbal consent to blood sampling was considered adequate by the local Ethics Committee (De Videnskabsetiske Komitéer for Region Hovedstaden), and was obtained. Biological specimens and all data obtained from their use for research were anonymized. Communication of research data among co-investigators in Copenhagen and Pittsburgh are also done in an anonymized manner in accordance with an exempt protocol approved by the Institutional Review Board of the University of Pittsburgh. Recruitment, verbal consent, and storage/use of blood specimens were carried out in accordance with the Declaration of Helsinki.

### Cell culture

The adult human RPE cell line ARPE-19 (American Type Culture Collection) was cultured in Dulbecco's Modified Eagle's Medium (DMEM) with 10% fetal calf serum, 300 µg/ml L-glutamine (Gibco), 50 µg/ml gentamicin (Gibco), and 2.5 µg/ml amphotericin B (Gibco), at 37°C with 10% CO_2_ for at least 6 weeks before use.

### Preparation of peripheral blood mononuclear cell (PBMC)-conditioned media (PCM)

PBMCs were isolated by isopycnic centrifugation (Lymphoprep™, Axis-Shield). PBMCs were cultured in DMEM in T75 flasks (Nunc) at 37°C with 5% CO_2_ for 1 week. Dynabeads® CD3/CD28 T Cell Expander (Invitrogen) were added at the beginning of the culture to activate T cells. The culture supernatant was harvested after 2, 4, and 7 days, and replaced with fresh medium containing 20 U/ml IL2 (Aldesleukin, Novartis). Conditioned medium was frozen immediately at −20°C. Flow cytometry analyses of PBMCs before and after culture showed expansion and activation of primarily T cells (data not shown). For RPE experiments, 50% of pooled PCM was used, and the culture medium with PCM was changed every 2–3 days. Replicates were performed using at least two different batches of PCM.

### MTT (3-(4,5-Dimethylthiazol-2-yl)-2,5-Diphenyltetrazolium Bromide) assay

RPE cells plated in 96-well plates were treated with (1) 50% PCM in fresh DMEM for 1–10 days; (2) 50% PCM in DMEM for 5 days followed by 1–9 days recovery with 100% fresh DMEM; (3) 0–1 mM sodium iodate (NaIO_3_) with or without 50% PCM for 24 h; (4) 0–2 mM hydrogen peroxide (H_2_O_2_) with or without 50% PCM for 24 h; and (5) 200 ng/ml IFNγ, and/or 20 ng/ml TNFα (both from R&D Systems) for 48 h. At the end of incubation, cell viability was assayed using the Vibrant® MTT Cell Proliferation Assay Kit (Molecular Probes). Briefly, cells were incubated with MTT labeling reagent for 3 h, and solubilization solution (sodium dodecyl sulfate (SDS) in HCl) for 18 h, before measuring absorbance at 560 nm on a VersaMax microplate reader (Molecular Devices). For each setup, 3–4 independent assays were performed, with 4–6 replicates per plate.

### ELISA

Secreted cytokines were quantified by sandwich ELISA as previously described [40].

### Light microscopy

RPE cells plated in 6-well plates were treated with fresh medium, 2 mM H_2_O_2_, or 1 mM NaIO_3_ for 24 h alone or together with 50% PCM, 200 ng/ml IFNγ, 20 ng/ml TNFα, or IFNγ+TNFα. Additionally, cells were treated with 50% PCM for 48 h with addition of 2 mM H_2_O_2_ or 1 mM NaIO_3_ for the last 24 h. At the end of incubation, one representative micrograph per well was taken using a Leica DM IRB inverted microscope equipped with a Leica Modulation Contrast (LMC) 10 objective, and a Leica DC300 camera using the Leica Image Manager (IM) 50 software. For each setup, three replicates were performed.

### Immunofluorescence microscopy

RPE cells plated on microscope cover slides were treated for 24 h with 1.5 mM H_2_O_2_ with or without 50% PCM in fresh DMEM. Cells to be stained with mouse anti-p62 were fixed with 2% paraformaldehyde in PBS for 30 min. at RT and permeabilized by Triton X-100 (0.2% in PBS with 1% BSA) for 15 min. at RT. Samples were blocked using TBS/0.05% Tween-20 with 3% skim milk for 1 h at RT. The mouse anti-SQSTM1/p62 (Abcam) was diluted 1∶100 in TBS/0.05% Tween-20, and cover slides were incubated overnight at 4°C. After washing with TBS/0.05% Tween-20, the cover slides were incubated with secondary Dy-Light 650-conjugated donkey anti-mouse antibody (Abcam) diluted 1∶50 in TBS/0.05% Tween-20 for 2 h at RT and washed. Hoechst 33258 was used to stain the nuclei. For visualization of mitochondrial transmembrane potential, cells were stained with JC-1 dye MitoGLO™ (Imgenex) using manufacturer's recommendations. 5 images per slide were captured using a Zeiss LSM 710 on Axio Imager.

### Flow cytometry

RPE cells plated in 6-well plates were incubated for 48 h with 50% PCM in fresh DMEM, or 100 ng/ml IFNγ and 10 ng/ml TNFα. For the last 24 h, 1.5 mM H_2_O_2_ or 0.6 mM NaIO_3_ was added. Cells were brought into single-cell suspension by 20–30 min incubation with 0.05% trypsin (Gibco). To quantify intracellular proteins p62 and caspase-3, cells were permeabilized with Cytofix/Cytoperm™ Fixation/Permeabilization Solution Kit (BD Pharmingen) before staining. The following stains and antibodies were used: anti-p62 and JC-1 were as described for the immunofluorescence assay above, FITC-conjugated Annexin V and propidium iodide (PI) (both from Biolegend), PE-conjugated rabbit-anti-active caspase-3 (BD Pharmingen), and APC-conjugated mouse-anti-HLA-ABC (BD Pharmingen). Isotype controls were PE-conjugated rabbit IgG (Santa Cruz) and APC-conjugated mouse IgG (BD Pharmingen). Manufacturer's recommendations for buffers and reagent amounts were followed. Flow cytometry was performed on a FACSCalibur cytometer (BD Biosciences), the isotype controls used for machine calibration to optimize fluorescence signals. Cytometric data were analyzed offline using FlowJo software (Tree Star Inc.). FSC/SSC plots were used to electronically gate out debris from whole cells. For Annexin V and JC-1, electronic gate of viable cells was established from the initial analysis of untreated cell controls, and signals from this gate were analyzed in accordance with manufacturer's recommendations.

### Microarrays

RPE cells were cultured alone, with CD3/CD28-activated T cells, or with recombinant human cytokines as previously described [40], [41]. Two genome-wide microarray types from Affymetrix, Human Genome U133 Plus 2.0 and Human Gene 1.0 ST were labeled and analyzed as previously described [40], [41]. Though the experimental setup (RPE:T cell co-culture system) for these microarrays differed from the rest of the study (using conditioned medium), the observed upregulation was validated at the protein level using conditioned medium.

### Immunoblotting

The antioxidative enzyme SOD2 was detected by immunoblotting of RPE cell lysates as previously described [41]. The antibodies used were mouse-anti-SOD2 (0.5 µg/ml, R&D Systems), horseradish-peroxidase (HRP)-conjugated, mouse-anti-beta-actin (1∶1000, Cell Signaling), and HRP-conjugated donkey anti-mouse IgG (1∶1000, R&D Systems). Blots were quantified using ImageJ software [42] following the method outlined at http://www.lukemiller.org/journal/2007/08/quantifying-western-blots-without.html.

### Statistics

Statistical analyses were performed using GraphPad Prism (GraphPad Software). Data from MTT assays were normalized to controls and analyzed with 1-way ANOVA with Tukey's multiple comparison. Flow cytometry data were analyzed with 1-way ANOVA with Tukey's multiple comparison. Immunoblot densitometry was analyzed by Student's *t*-test. P-values less than 0.05 were considered significant.

## Results

### Reduced viability of RPE cells after treatment with PCM

**Figure 1:**
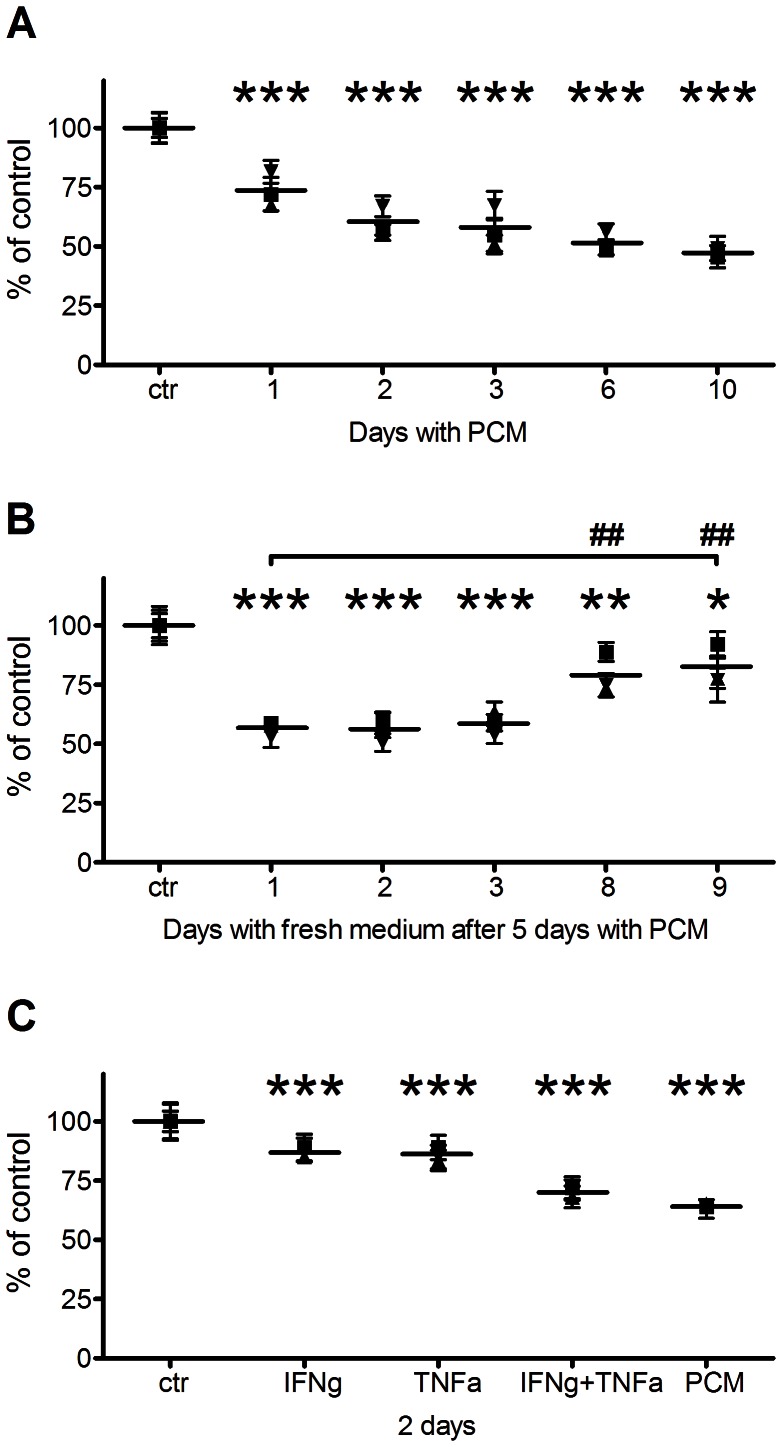
Effects of inflammatory cytokines on RPE cell viability. MTT assay data from RPE cells cultured in (A) 50% PCM with 50% fresh medium for 1–10 days, (B) 50% PCM for five days, and subsequently in 100% fresh medium for 1–9 days, and (C) 200 ng/ml IFNγ and/or 20 ng/ml TNFα, or 50% PCM for 48 hours. Ctr, RPE cells cultured in fresh medium. Data are shown as mean of each experiment with error bars indicating SD, and a vertical line indicating mean of 3 experiments. *, P<0.05; **, P<0.01; ***, P<0.001 vs. control. ##, P<0.01 vs. day 1.

To investigate RPE cell reaction to a mixture of inflammatory cytokines from CD3/CD28-activated PBMCs, RPE cells were incubated for 1–10 days with 50% PCM and 50% fresh medium to ensure adequate amounts of nutrients. Cell viability was monitored with the MTT assay and normalized to untreated cells. Because the RPE cells were used at high confluence, the MTT assay recorded the level of cell survival, not cell proliferation. RPE cell viability was significantly decreased after 24 hours' exposure to PCM, and viability further decreased in a time-dependent manner during day 2–10. After 10 days' PCM exposure, only 50% of RPE cells were viable (Figure 1A). While the reduction in viability could potentially be due to the reduced growth factors in the medium containing 50% PCM, our empirical studies have shown that dense, non-dividing RPE cultures may survive months in the total absence of serum. To determine if RPE cell viability could be recovered, cells were incubated with 50% PCM for 5 days before changing back to 100% fresh medium. Cell viability was quantified with the MTT assay after culture in fresh medium for 1–9 days, and normalized to untreated cells. Viability was significantly reduced during the entire follow-up period, but after 8–9 days of fresh medium, cell viability was significantly higher than after 1 day of fresh medium, indicating that the RPE cells recovered and proliferated (Figure 1B).

### Reduced viability of RPE cells after treatment with IFNγ and TNFα

Based on previous microarray studies of gene expression of cytokines and cytokine receptors in activated T cells co-cultured with RPE cells, we had identified IFNγ and TNFα as candidate effectors of T cell-related changes in RPE phenotypes [40]. To study the direct effect(s) of these two cytokines on RPE cell viability, RPE cells were incubated for 48 hours with 200 ng/ml IFNγ and/or 20 ng/ml TNFα; concentrations comparable to those found in the employed PCM (data not shown). Cell viability was monitored with the MTT assay. IFNγ and TNFα each caused a 13–14% decrease in cell viability. These individual cytokine effects were additive and cell viability decreased with 30% when both IFNγ and TNFα were present (Figure 1C).

### RPE cell death by oxidative agents is attenuated by PCM exposure

**Figure 2:**
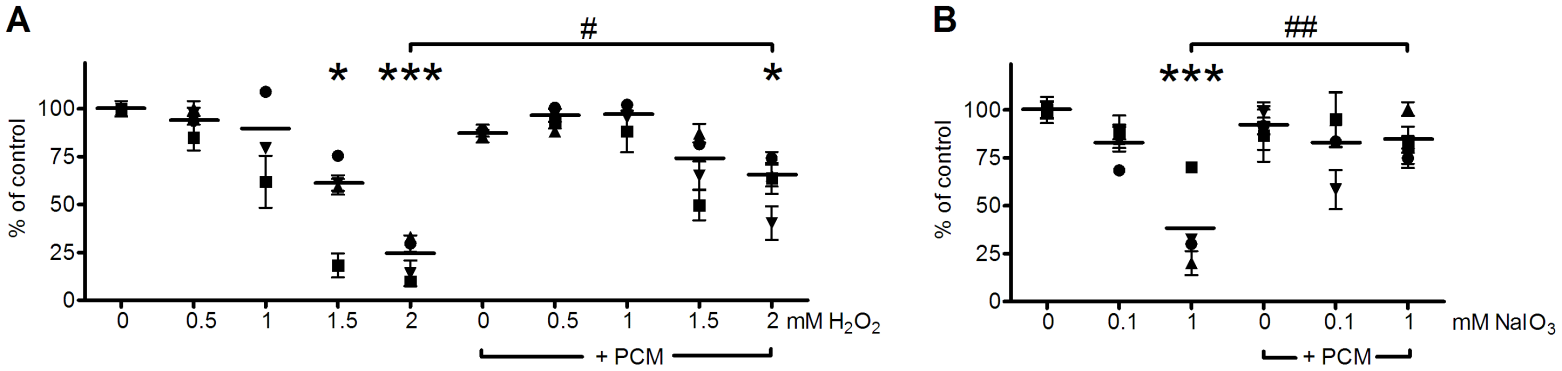
PCM rescues RPE cells from NaIO_3_ or H_2_O_2_-induced death. MTT assay data from (A) RPE cells cultured with 0–2 mM H_2_O_2_ for 24 hours with or without 50% PCM; (B) RPE cells cultured with 0–1 mM NaIO_3_ for 24 hours with or without 50% PCM; Ctr, RPE cells cultured in fresh medium. Data are shown as mean of each experiment with error bars indicating SD, and a vertical line indicating mean of 3 experiments. *, P<0.05; ***, P<0.001 compared to control. #, P<0.05; ##, P<0.01 compared to oxidative agent alone.

To examine the effect of treatment with oxidative agents, RPE cells were cultured for 24 hours with different concentrations of NaIO_3_ or H_2_O_2_ alone, or in combination with 50% PCM. Cell viability was monitored with the MTT assay. At 1.5 and 2 mM H_2_O_2_ cell viability was significantly reduced. When both H_2_O_2_ and PCM were added, RPE cells were significantly protected from cell death compared to cultures treated with 2 mM H_2_O_2_. However, PCM could not completely restore viability to the level of untreated cells (Figure 2A). Similarly, 1 mM NaIO_3_ significantly reduced cell viability. When both 1 mM NaIO_3_ and PCM was added, cells were significantly more viable than cells treated with 1 mM NaIO_3_ alone, and viability was not significantly different from untreated cells (Figure 2B).

### Prevention of RPE cell death by oxidative agents is in part mediated by IFNγ and TNFα

**Figure 3:**
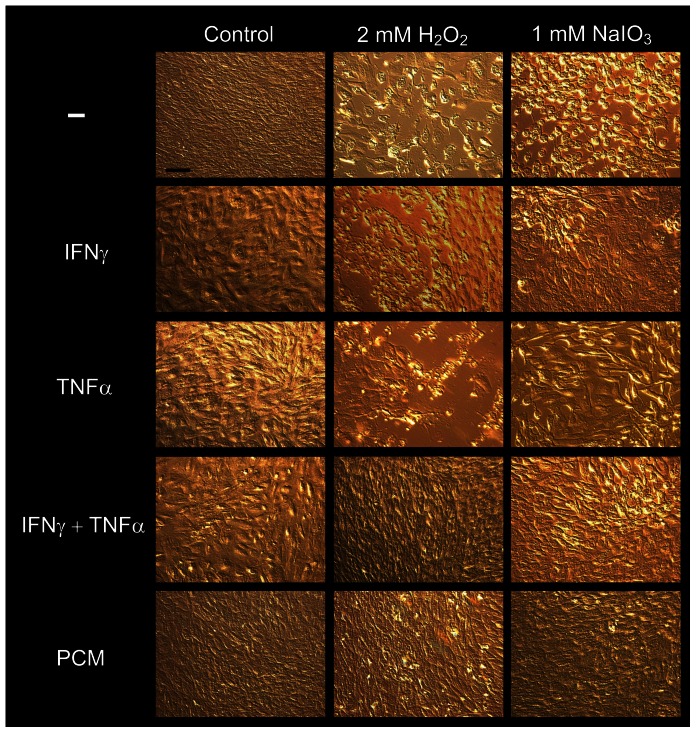
RPE cells die from exposure to oxidative agents, but IFNγ and TNFα reduce the level of NaIO_3_/H_2_O_2_-induced death. Micrographs of RPE cells cultured in fresh medium (Ctr) or with 2 mM H_2_O_2_ or 1 mM NaIO_3_ for 24 hours, alone or with IFNγ, TNFα, IFNγ+TNFα, or 50% PCM added. All micrographs were taken at 100× magnification and are representative of the entire well. At least three replicates were performed for each treatment. Black bar in upper left image, 100 µm.

Our previous studies on RPE cultured with T cell-derived humoral factors have revealed IFNγ and TNFα as the major effector cytokines [40]. We therefore investigated whether the observed protective effect of PCM was due to IFNγ and TNFα. RPE cells were treated with H_2_O_2_, NaIO_3_, PCM, IFNγ, TNFα, or combinations of these. After 24 hours, cells were examined microscopically, and representative micrographs were recorded. Untreated cells formed a tightly packed monolayer with ordered cobblestone appearance (Figure 3). Treatment with cytokines or PCM resulted in a flattened, less orderly appearance, presumably due to spreading of surviving cells into areas vacated by dead cells. There was no significant morphological differences between the four inflammatory treatments. In wells treated with H_2_O_2_ or NaIO_3,_ there were large areas of dead cells, and remaining cells were rounded. Concomitant addition of IFNγ or TNFα to cells treated with oxidative agent resulted in reduced areas of dead cells. However, addition of IFNγ+TNFα or PCM together with either H_2_O_2_ or NaIO_3_ resulted in markedly reduced areas of cell death.

### Oxidative agents elicit mitochondrial dysfunction and inhibit autophagy, both of which are reversed by IFNγ and TNFα

**Figure 4:**
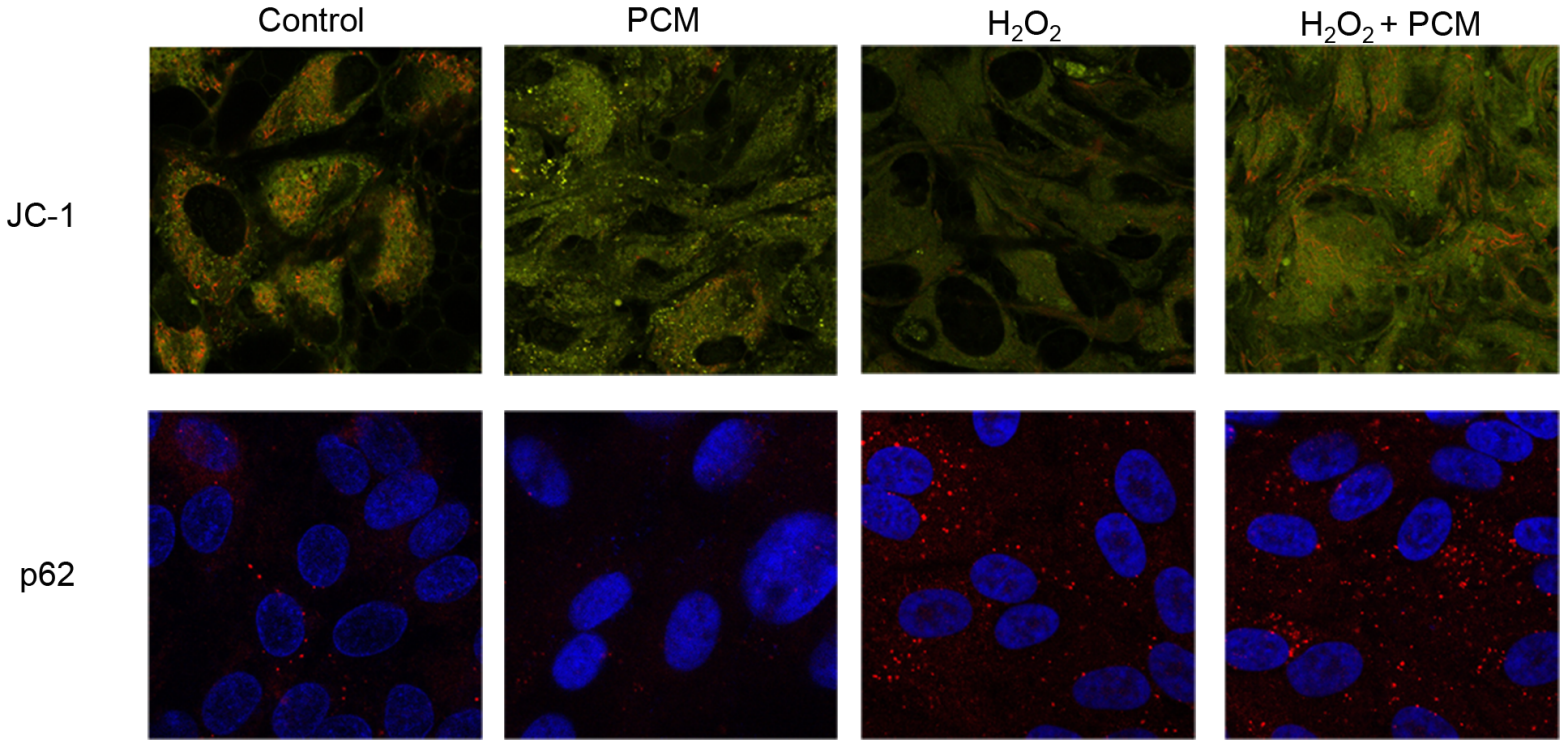
H_2_O_2_ induces mitochondrial depolarization and increases intra-organelle p62. Immunofluorescence micrographs from RPE cells cultured for 24 hours with 50% PCM and/or 2 mM H_2_O_2_. (Top) Cells were stained with JC-1. The *orange color* represents aggregated JC-1 indicative of functional, polarized mitochondria. The *green color* represents JC-1 monomers found throughout the cytosol of all cells. (Bottom) Permeabilized cells stained with anti-p62 (*red puncta*), with nuclei counterstained with Hoechst (*blue*).

To further investigate the cellular changes leading to cell death, RPE cells were cultured on cover slips and treated with H_2_O_2_ and/or PCM for 24 hours. The mitochondrial dye JC-1 was used to visualize mitochondrial function. JC-1 fluoresces green as a monomer, and red as an aggregate; the latter is a bioindicator of the high transmembrane potential of functional polarized mitochondria [43]. Results showed that aggregated JC-1 was found in elongated structures throughout the cytoplasm of untreated cells as well as cells treated with PCM or H_2_O_2_+PCM (Figure 4), and JC-1 aggregates were diminished in cells treated with H_2_O_2_ alone. In cells treated with PCM alone, the aggregated JC-1 co-localized with puncta of intense monomer (green) staining, giving the mitochondria a yellow color on the overlay. p62 (Sequestosome-1, SQSTM1) is a key autophagic protein, which localizes to the lysosomes during autophagy [44]. In untreated RPE cells, p62 appeared as puncta in the cytoplasm, and the number of puncta greatly increased with H_2_O_2_ treatment in the presence or absence of PCM (Figure 4). Since p62 is consumed in the autophagic process [45], this finding suggested a H_2_O_2_-induced inhibition of autophagy.

**Figure 5:**
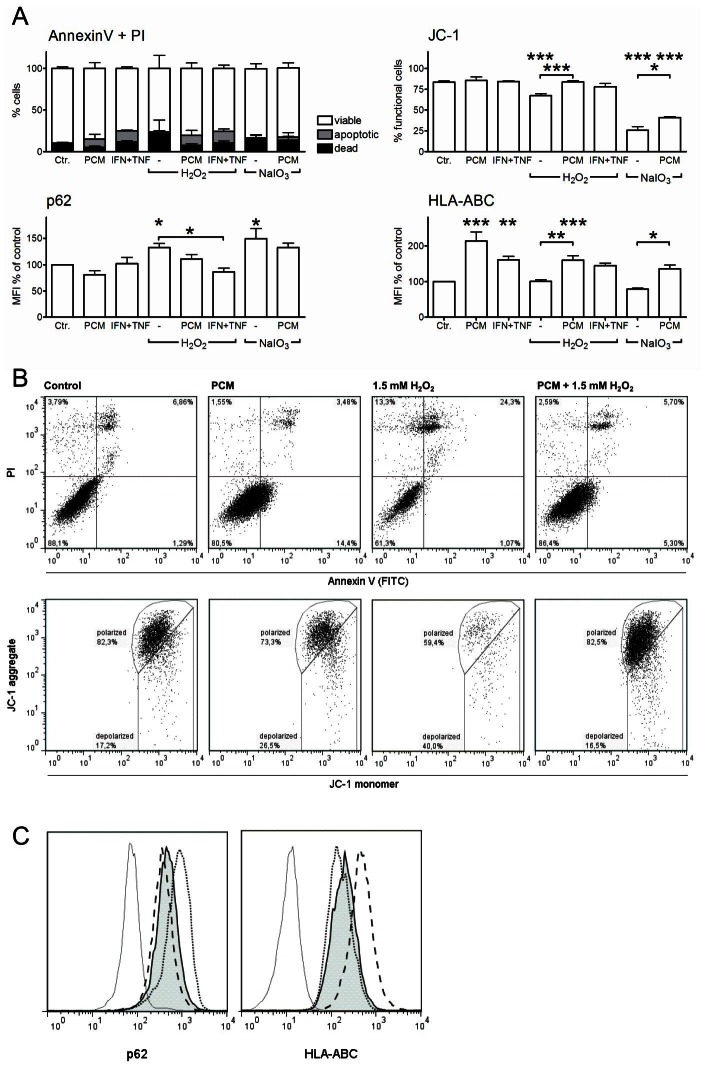
IFNγ and TNFα protect RPE from cell death from oxidative agents through normalization of mitochondrial function and autophagy. Flow cytometric analyses of RPE cells treated with PCM or IFNγ+TNFα for 48 hours, with addition of 1.5 mM H_2_O_2_ or 0.6 mM NaIO_3_ for the last 24 hours. (A) Bar graphs of data showing mean of 3–6 replicates with error bars indicating SD. *, P<0.05; **, P<0.01; ***, P<0.001 compared to control. (B) For Annexin V/PI and JC-1, data are shown as % cells in the relevant gates shown in representative scatter plots. (C) For p62 and HLA-ABC, data are shown as % of control median fluorescent intensity (MFI), and representative histograms are shown in. The peaks shown in (C) are: isotype (hairline), control (tinted peak), PCM (dashed line), and NaIO_3_ (dotted line).

To quantify the observed changes in mitochondrial function and autophagy, we cultured RPE cells with lower concentrations of H_2_O_2_ or NaIO_3_ with or without pretreatment with PCM or IFNγ + TNFα. Cells were analyzed using flow cytometry. After incubation with H_2_O_2_ or NaIO_3_, we observed some dead non-adherent cells that were removed before trypsination and staining. In the remaining adherent cells, there was no statistically significant decrease in cell viability in any of the treatments (Annexin V/PI staining, Figure 5A and B). There was a tendency towards an increase in the percentage of early apoptotic cells (Annexin V-positive and PI-negative) after treatment with PCM or IFNγ + TNFα, and towards a slight increase in the percentage of dead cells (PI-positive) after treatment with H_2_O_2_ or NaIO_3_. We detected no Caspase-3 activation in any treatment groups (data not shown). Mitochondrial transmembrane potential was significantly decreased in cells treated with H_2_O_2_ or NaIO_3_. Pretreatment with PCM normalized mitochondrial transmembrane potential (JC-1 staining, Figure 5A and B). Treatment with H_2_O_2_ or NaIO_3_ increased intracellular p62, and pretreatment with PCM tended to decrease p62 levels, while IFNγ + TNFα normalized p62 for H_2_O_2_-treated cells (Figure 5A and C). As an internal control, HLA-ABC (pan-major histocompatibility complex 1) that is constitutively expressed on all cells was upregulated by IFNγ as expected (Figure 5A and C).

### Upregulation of anti-oxidative genes and proteins in RPE cells after co-culture with T cells

**Figure 6:**
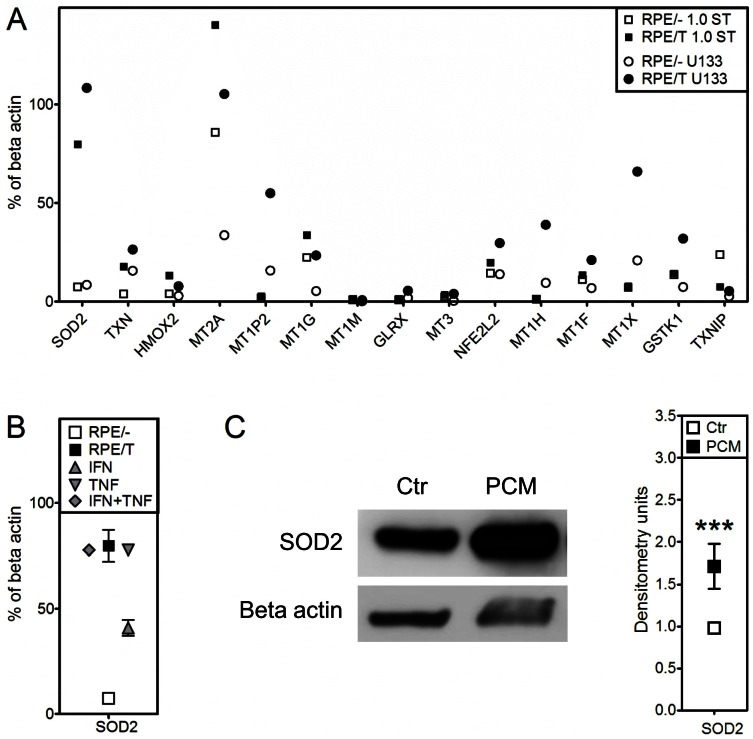
RPE cells exposed to cytokines upregulate expression of anti-oxidative genes and SOD2 protein. (A+B) Microarray analyses of RPE cells cultured in a transwell system with activated purified T cells, or with IFNγ+/-TNFα added to the basolateral side. (A) Gene expression was analyzed using two different microarray chips, Human Genome U133 Plus 2.0 and Human Gene 1.0 ST. Data are shown as the mean of % of beta actin expression for two replicates for each chip, for genes upregulated >2-fold compared with control. (B) SOD2 gene expression was analyzed with Human Gene 1.0 ST, and data shown as mean of % of beta actin expression with error bars indicating SD. (C) Immunoblot of SOD2 of RPE cell lysates treated with 50% PCM. A representative blot is shown, and densitometry performed on two replicates. Data are shown as mean densitometry units (± SD bars) normalized to β-actin. ***, P<0.001.

To elucidate mechanism(s) of protection from oxidative stress by T cell-derived humoral factors, we purified T cells from whole blood using antibody-based negative selection as previously described [41]. CD3/CD28-activated T cells were added basolaterally to RPE cells in a transwell system, and RPE gene expression examined by microarrays. A total of 97 genes related to the anti-oxidant stress response were identified by literature search (Table S1). Genes upregulated more than 2-fold by co-culture with T cells in either of the two microarrays (U133 plus 2.0 or 1.0 ST) are shown in Figure 6A. The mitochondrial form of superoxide dismutase (SOD2) was highly expressed, and further upregulated after T cell co-culture for both arrays. Other genes showing similar baseline and upregulated expression levels in the two arrays were thioredoxin (TXN), metallothioneins MT1G and MT2A, heme oxygenase 2 (HMOX2), and the master regulator of the anti-oxidative stress response, nuclear factor (erythroid-derived 2)-like 2 (NFEL2 also known as NFE2-related factor, Nrf2).

To test whether IFNγ and/or TNFα are components of the RPE-activating T cell culture supernatants, we performed microarrays on RPE cells treated with either or both of these cytokines. IFNγ alone increased expression of SOD2 partly, but TNFα alone or with IFNγ induced SOD2 expression to the same level as that induced by T cell co-culture. Immunoblots were performed on RPE cell lysates, to examine whether SOD2 protein expression was also increased after treatment with PCM. Treatment with PCM increased SOD2 protein expression significantly (Figure 6C).

## Discussion

In this study, we found that treatment with PCM, IFNγ, TNFα, H_2_O_2_, or NaIO_3_ decreased RPE cell viability. However, concomitant treatment with PCM and oxidative agents protected from cell death, possibly through induction of autophagic and anti-oxidative pathways. In a previous microarray study with RPE cells co-cultured with purified activated T cells, we reported that IFNγ and TNFα are the main cytokines mediating cellular crosstalk in this culture system [40]. In the present work, we found that IFNγ and TNFα alone account for part of the protection due to PCM. Identity of other humoral factors in PCM augmenting this protection remains to be examined.

### Oxidative damage and mitochondrial stress in the RPE

NaIO_3_ is a known oxidative agent of RPE cells [46]. Other studies have also shown that H_2_O_2_, another oxidative agent, induces RPE cell death characterized by the production of ROS [47]–[49], cytochrome *c* release [50], [51], DNA fragmentation [49], [51], Annexin V-binding [52], TUNEL-staining [50], and caspase-3 activation [47], [49], [51], [53]. RPE cells can be protected from H_2_O_2_-induced cell death by treatment with antioxidants such as ascorbate (vitamin C) [54], N-acetylcysteine [47], and quercetin [55]. These studies indicate that H_2_O_2_ causes cell death in RPE cells through mitochondrial dysfunction, ROS production, and induction of apoptosis. In the present work, we found that mitochondrial depolarization preceded RPE cell death due to NaIO_3_ and H_2_O_2_, and that PCM and IFNγ+TNFα protected the cells from mitochondrial destabilization. Mitochondrial dysfunction as a result of oxidative damage could lead to in diminished energy production, cytochrome *c* release, and ROS production, all of which contribute to cellular stress and ultimately lead to apoptosis [56]. High numbers of mitochondria are present in metabolically active cells like the RPE cells, but mitochondrion density decreases with age, particularly in AMD eyes [57], [58]. Mitochondria are especially exposed to oxidative stress because of the high ROS production by the respiratory chain and their less efficient DNA repair systems [25]. Indeed, mitochondrial DNA damage has been shown to increase in RPE cells corresponding with age and AMD status [25], [59], and RPE from AMD patients had decreased anti-oxidative capacity [60].

### Oxidative stress, inflammation, and regulation of autophagy

Our data show decreased viability of RPE cells after treatment with IFNγ and/or TNFα. Previous studies have reported TNFα to have a growth-stimulating effect on sub-confluent cultures (80% confluency; </ = 100,000 cells/cm^2^) of primary human or bovine RPE cells, while dense cultures were unaffected or inhibited by TNFα [61], [62]. It is likely that the observed growth-stimulating effect of TNFα on RPE cells is only present in actively dividing cultures, and/or on primary cells. Thus, these findings do not necessarily conflict with our results, since we used dense, non-dividing (>180,000 cells/cm^2^) cultures of ARPE-19 cells. Our data also show that p62 accumulated in RPE cells following treatment with H_2_O_2_ or NaIO_3_. p62 (also known as sequestosome 1) is a key autophagic protein linking the proteasomal and lysosomal clearance systems through its interaction with ubiquitin [32], [39], [44]. Autophagy is a dynamic process, and can only be measured accurately by autophagic flux, not by the amount of autophagy-related proteins at a given time point [63]. Since p62 is consumed in the autophagic process, an increase in intracellular p62 can be the result of increased p62 production and/or blocked autophagy [45]. In two studies of ARPE-19 cells, treatment with autophagy inhibitors bafilomycin A or chloroquine increased intracellular p62 levels [64], [65]. Oxidative stress surpassing the anti-oxidative capacity of a cell results in the damage of proteins and mitochondria, thereby increasing the need for effective autophagy. However, oxidative stress can also inhibit autophagy by damaging lysosomal membranes [66]. A study of ARPE-19 cells exposed to H_2_O_2_ indeed showed a decrease in the autophagic flux [67]. These observations indicate that in ARPE-19 cells, inhibition of autophagy results in increased p62 levels, and H_2_O_2_ reduces the efficiency of the autophagic process. In the present study, we found that oxidative agents increased intracellular p62 levels. Pretreatment with IFNγ+TNFα normalized p62 levels, indicating that the combined treatment with these two cytokines could be increasing autophagy in resting and oxidatively stressed RPE cells.

### Inflammation and protection against oxidative stress

Inflammation is known to be involved in AMD pathogenesis. We have recently shown that co-culture with activated T cells increases complement [41] and chemokine [40] expression in RPE cells, potentially augmenting inflammation in the early AMD retina. However, our whole-transcriptome analysis showed increased gene expression of anti-oxidative enzymes in response to TNFα and IFNγ. This suggested that the RPE cell response to inflammatory stress also includes protective mechanisms. In the present work, we found increased expression of the anti-oxidative enzyme SOD2 in RPE cells exposed to PCM. SOD2 is an important mitochondrial anti-oxidative enzyme, catalyzing superoxide ion conversion to H_2_O_2_ and oxygen. The resulting H_2_O_2_ is degraded by catalase and glutathione peroxidases [68]. In vitro and in vivo studies report that SOD2 expression protects RPE cells from H_2_O_2_-induced oxidative stress [50], [69]. Elner and co-workers found that co-culture with monocytes induced superoxide production, causing mitochondrial depolarization and apoptosis in murine RPE cells. This was especially pronounced in RPE cells from *Sod2* ^+/−^ mice [70].

Other components of the anti-oxidative stress response are likely important for RPE cell survival. The transcription factor NFE2L2 (alias NFE2-related factor, Nrf2) is the master regulator of the anti-oxidative stress response. Nrf2 activation protects against oxidative stress and reduces inflammation in the eye, but the Nrf2 response is reduced with aging [24]. The importance of Nrf2 was demonstrated using *Nrf2* ^−/−^ mice that spontaneously developed sub-RPE deposits containing inflammatory proteins and RPE pathology within 12 months of age [71]. In this study, we found increased gene expression of NFE2L2 in RPE cells co-cultured with activated T cells, further supporting the idea that inflammation elicits a protective anti-oxidative response in RPE cells.

### Conclusion

In this study, we found that inflammatory mediators protect RPE cells from oxidative stress-mediated cell death, by upregulating the anti-oxidative pathway that includes SOD2 enzyme, and by inhibiting mitochondrial stress and promoting autophagy. Several anti-inflammatory drugs are currently undergoing clinical testing for use in AMD, including complement inhibitors, corticosteroids, NSAIDs, and immunosuppressants [18], [20], [72]. Our data suggest caution about the use of therapies aimed at down-regulating the retinal immune response, since they could have an unintended adverse cellular effect for increased sensitivity to oxidative stress.

## Supporting Information

Table S1 **RPE cell expression of anti-oxidant stress response genes.** Gene expression in RPE cells of 97 genes identified by a literature search to be related to the anti-oxidant stress response. The table shows expression of target gene as per cent of beta actin (ACTB) expression, for microarrays Gene 1.0 ST and U133 plus 2.0. RPE/- denotes untreated RPE cells; RPE/T denotes RPE cells co-cultured with CD3/CD28-activated T cells added basolaterally to the RPE cells in a transwell system for 48 hours.
(PDF)
