# Supplementary material for: Inflammatory Cytokines Protect Retinal Pigment Epithelial Cells from Oxidative Stress-Induced Death
Source: PLoS One. 2013 May 21;8(5):e64619. doi: 10.1371/journal.pone.0064619 (PMC3660526; doi:10.1371/journal.pone.0064619)
Supplement: Table S1 — RPE cell expression of anti-oxidant stress response genes. Gene expression in RPE cells of 97 genes identified by a literature search to be related to the anti-oxidant stress response. The table shows expression of target gene as per cent of beta actin (ACTB) expression, for microarrays Gene 1.0 ST and U133 plus 2.0. RPE/- denotes untreated RPE cells; RPE/T denotes RPE cells co-cultured with CD3/CD28-activated T cells added basolaterally to the RPE cells in a transwell system for 48 hours. (PDF) [file pone.0064619.s001.pdf]

Gene Symbol	Gene Accession	Gene Description	Gene 1.0 ST Probe Set ID	Gene expression, % of ACTB RPE/-	RPE/T	U133 plus 2.0 Probe Set ID	Gene expression, % of ACTB RPE/-	RPE/T
ATF4	NM_001675	activating transcription factor 4	8073148	11	11	222103_at	3	3
BLVRA	NM_000712	biliverdin reductase A	8132515	2	2	211729_x_at	5	10
BLVRB	NM_000713	biliverdin reductase B	8036913	3	3	202201_at	5	5
CAT	NM_001752	catalase	7939298	9	4	201432_at	20	11
FTH1	NM_002032	ferritin, heavy polypeptide 1	8170360	125	131	200748_s_at	127	172
FTL	NM_000146	ferritin, light polypeptide	8030171	24	20	212788_x_at	87	72
GLO1	NM_006708	glyoxalase I	8126135	12	11	200681_at	21	15
GLRX	NM_002064	glutaredoxin (thioltransferase)	8113214	1	1	206662_at	2	5
GLRX2	NM_197962	glutaredoxin 2	7923027	1	2	219933_at	5	5
GLRX3	NM_006541	glutaredoxin 3	7931393	3	3	209080_x_at	9	6
GLRX5	NM_016417	glutaredoxin 5	8169709	5	4	221932_s_at	4	2
GPX1	NM_201397	glutathione peroxidase 1	8087405	11	9	200736_s_at	60	30
GPX2	NM_002083	glutathione peroxidase 2	7979658	1	1	202831_at	0	0
GPX3	NM_002084	glutathione peroxidase 3	8109333	28	8	201348_at	35	14
GPX4	NM_002085	glutathione peroxidase 4	8024194	64	25	201106_at	35	15
GPX5	NM_001509	glutathione peroxidase 5	8117700	0	0	208028_s_at	0	0
GPX6	NM_182701	glutathione peroxidase 6	8124588	0	0			
GPX7	NM_015696	glutathione peroxidase 7	7901460	1	1	213170_at	1	1
GPX8	NM_001008397	glutathione peroxidase 8	8105348	5	10			
GSR	NM_000637	glutathione reductase	8150112	15	10	225609_at	2	1
GSTA1	NM_145740	glutathione S-transferase alpha 1	8127072	0	0	215766_at	1	0
GSTA2	NM_000846	glutathione S-transferase alpha 2	8127065	0	0	242478_at	0	0
GSTA3	NM_000847	glutathione S-transferase alpha 3	8127087	0	0	222102_at	0	0
GSTA4	NM_001512	glutathione S-transferase alpha 4	8127094	4	1	202967_at	3	2
GSTA5	NM_153699	glutathione S-transferase alpha 5	8127079	0	0			
GSTCD	NM_001031720	glutathione S-transferase, C-terminal domain containing	8096688	1	1	235387_at	1	0
GSTK1	NM_001143679	glutathione S-transferase kappa 1	8136849	14	14	217751_at	7	32
GSTM1	NM_000561	glutathione S-transferase mu 1	7903765	14	3	215333_x_at	3	1
GSTM2	NM_000848	glutathione S-transferase mu 2	7903753	7	1	204418_x_at	5	2
GSTM2P1	NR_002932	glutathione S-transferase mu 2 pseudogene 1	8128890	1	1			
GSTM3	NM_000849	glutathione S-transferase mu 3	7918379	2	2	202554_s_at	11	11
GSTM4	NM_000850	glutathione S-transferase mu 4	7903742	12	1	204149_s_at	1	0
GSTM5	NM_000851	glutathione S-transferase mu 5	7903777	1	0	205752_s_at	1	0
GSTO1	NM_004832	glutathione S-transferase omega 1	7930304	2	5	201470_at	27	35
GSTO2	NM_183239	glutathione S-transferase omega 2	7930311	3	2	227163_at	2	0
GSTP1	NM_000852	glutathione S-transferase pi 1	7941936	56	29	200824_at	30	14
GSTT1	NM_000853	glutathione S-transferase theta 1	8074980	4	2	203815_at	1	1
GSTT2	NM_000854	glutathione S-transferase theta 2	8071809	4	3	205439_at	1	2
GSTTP1	NR_003081	glutathione S-transferase theta pseudogene 1	8071801	0	0	207215_at	0	0
GSTZ1	NM_001513	glutathione transferase zeta 1	7975956	2	1	209531_at	1	1
HMOX1	NM_002133	heme oxygenase (decycling) 1	8072678	8	10	203665_at	2	2
HMOX2	NM_001127204	heme oxygenase (decycling) 2	7992987	4	13	218120_s_at	3	8
MGST1	NM_145792	microsomal glutathione S-transferase 1	7954196	14	10	224918_x_at	44	42
MGST2	NM_002413	microsomal glutathione S-transferase 2	8097513	4	1	204168_at	5	3
MGST3	NM_004528	microsomal glutathione S-transferase 3	7906978	24	25	201403_s_at	21	18
MPO	NM_000250	myeloperoxidase	8016932	1	1	203948_s_at	0	0
MT1A	NM_005946	metallothionein 1A	7995806	1	2			
MT1B	NM_005947	metallothionein 1B	7995820	1	2			
MT1DP	NR_027781	metallothionein 1D (pseudogene)	7995813	12	16			
MT1E	NM_175617	metallothionein 1E	7995797	9	11	212859_x_at	22	43
MT1F	NM_005949	metallothionein 1F	7995825	11	14	217165_x_at	7	21
MT1G	NM_005950	metallothionein 1G	8001531	22	34	204745_x_at	5	23
MT1H	NM_005951	metallothionein 1H	7995829	1	2	206461_x_at	9	39
MT1IP	NR_003669	metallothionein 1I (pseudogene)	7995834	1	2			
MT1JP	NR_036677	metallothionein 1J (pseudogene)	7995803	1	2	213629_x_at	3	6
MT1L	NR_001447	metallothionein 1L	7995793	9	12			
MT1M	NM_176870	metallothionein 1M	7995787	1	1	217546_at	0	1
MT1P2	AF333388	metallothionein 1 pseudogene 2	7925413	2	3	211456_x_at	16	55
MT1P3	BC103840	metallothionein 1 pseudogene 3	8062119	4	6	78047_s_at	5	7
MT1X	NM_005952	metallothionein 1X	7995838	7	8	208581_x_at	21	66
MT2A	NM_005953	metallothionein 2A	7995783	86	140	212185_x_at	34	105
MT3	NM_005954	metallothionein 3	7995776	2	3	205970_at	0	4
MT4	NM_032935	metallothionein 4	7995772	1	1	217395_at	0	0
NFE2L2	NM_006164	nuclear factor (erythroid-derived 2)-like 2	8056977	14	20	201146_at	14	30
PON1	NM_000446	paraoxonase 1	8141052	0	0	206345_s_at	0	0
PON2	NM_000305	paraoxonase 2	8141076	12	5	201876_at	15	9
PON3	NM_000940	paraoxonase 3	8141066	0	0	213695_at	0	0
PRDX1	NM_002574	peroxiredoxin 1	7915733	18	14	208680_at	29	16
PRDX2	NM_005809	peroxiredoxin 2	8034544	3	2	39729_at	32	15
PRDX3	NM_006793	peroxiredoxin 3	7936661	13	10	201619_at	25	16
PRDX4	NM_006406	peroxiredoxin 4	8166455	5	5	201923_at	45	50
PRDX5	NM_012094	peroxiredoxin 5	7940996	12	10	1560587_s_at	28	24
PRDX6	NM_004905	peroxiredoxin 6	7907439	17	9	200845_s_at	23	10
SOD1	NM_000454	superoxide dismutase 1, soluble	8068168	13	8	200642_at	43	37
SOD2	NM_001024465	superoxide dismutase 2, mitochondrial	8130556	7	80	215223_s_at	9	108
SOD3	NM_003102	superoxide dismutase 3, extracellular	8094372	16	10	205236_x_at	1	2
SQSTM1	NM_003900	sequestosome 1	8110569	18	24	201471_s_at	26	14
SRXN1	NM_080725	sulfiredoxin 1	8064375	3	3	225252_at	4	3
TXN	NM_003329	thioredoxin	8163185	4	18	208864_s_at	16	26
TXN2	NM_012473	thioredoxin 2	8075778	5	2	209077_at	4	2
TXNDC11	NM_015914	thioredoxin domain containing 11	7999478	7	5	223325_at	2	2
TXNDC12	NM_015913	thioredoxin domain containing 12	7916120	13	12	223017_at	13	10
TXNDC15	NM_024715	thioredoxin domain containing 15	8108166	7	5	220495_s_at	11	17
TXNDC16	NM_020784	thioredoxin domain containing 16	7979158	1	0	226747_at	2	0
TXNDC17	NM_032731	thioredoxin domain containing 17	8004175	1	1	224511_s_at	11	18
TXNDC2	NM_001098529	thioredoxin domain containing 2	8020123	1	1	1552657_a_at	0	0
TXNDC3	NM_016616	thioredoxin domain containing 3	8132349	0	0	220384_at	0	0
TXNDC6	NM_178130	thioredoxin domain containing 6	8090972	1	1	236966_at	0	1
TXNDC8	NM_001003936	thioredoxin domain containing 8	8163193	0	0	1564386_at	0	0
TXNDC9	NM_005783	thioredoxin domain containing 9	8054217	1	2	211758_x_at	7	6
TXNIP	NM_006472	thioredoxin interacting protein	7904726	24	7	201008_s_at	3	5
TXNL1	NR_024546	thioredoxin-like 1	8023450	5	6	201588_at	18	18
TXNL4A	NM_006701	thioredoxin-like 4A	8023920	7	9	202836_s_at	12	12
TXNL4B	NM_017853	thioredoxin-like 4B	8002660	2	3	218794_s_at	1	1
TXNRD1	NM_003330	thioredoxin reductase 1	7958174	5	6	201266_at	35	12
TXNRD2	NM_006440	thioredoxin reductase 2	8074498	2	2	211177_s_at	1	0
TXNRD3	NM_052883	thioredoxin reductase 3	8090395	1	1	59631_at	1	0
